# Cost analysis of depression using the national insurance system in South Korea: a comparison of depression and treatment-resistant depression

**DOI:** 10.1186/s12913-020-05153-1

**Published:** 2020-04-06

**Authors:** Daun Shin, Nam Woo Kim, Min Ji Kim, Sang Jin Rhee, Christopher Hyung Keun Park, Hyeyoung Kim, Bo Ram Yang, Mi-sook Kim, Gum Jee Choi, Minjung Koh, Yong Min Ahn

**Affiliations:** 1 Department of Neuropsychiatry, Seoul National University Hospital, 101 Daehak-ro, Jongno-gu, Seoul, 03080 Republic of Korea; 2 Department of Biomedical Sciences, Seoul National University Graduate school, 101 Daehak-ro, Jongno-gu, Seoul, 03080 Republic of Korea; 3 Department of Psychiatry and Behavioral Science, Institute of Human Behavioral Medicine, Seoul National University College of Medicine, 103 Daehak-ro, Jongno-gu, Seoul, 03080 Republic of Korea; 4 Department of Psychiatry, Asan Medical Center, 88 Olympic-ro 43-gil, Songpa-gu, Seoul, 05505 Republic of Korea; 5 Inha University Hospital, Neuropsychiatry, Incheon, South Korea; 6 Division of clinical epidemiology, Medical Research Collaborating Center, Seoul National University Hospital, Seoul, South Korea; 7 Medical Affairs, Janssen Korea, Seoul, South Korea

**Keywords:** Costs and cost analysis, Depression, Depressive disorder, treatment-resistant, National Health Programs

## Abstract

**Background:**

The incidence and burden of depressive disorders are increasing in South Korea. There are many differences between pharmaceutically treated depression (PTD) and treatment-resistant depression (TRD), including the economic consequences; however, to our knowledge, the economic burden of depression is understudied in South Korea. Therefore, the objective of the present study was to calculate the different economic costs of PTD and TRD in South Korea, specifically by comparing several aspects of medical care.

**Methods:**

This study comprised patients aged 18 and over who were newly prescribed antidepressants for more than 28 days with a depression code included from January 1, 2012, to December 31, 2012, by the Health Insurance Review and Assessment Service (HIRA). TRD was classified as more than two antidepressant regimen failures in PTD patients. The cost was calculated based on the cost reflected on the receipt registered with HIRA.

**Results:**

Of the 834,694 patients with PTD, 34,812 patients (4.17%) were converted to TRD. The cost of medical care for TRD (6,610,487 KRW, 5881 USD) was approximately 5 times higher than the cost of non-TRD (1,273,045 KRW, 1133 USD) and was significantly higher for patients with or without depression and suicide codes. Medical expenses incurred by non-psychiatrists were roughly 1.7 times higher than those incurred by psychiatrists.

**Conclusions:**

TRD patients had significantly higher healthcare costs than PTD patients. Identifying these financial aspects of care for depression can help to establish a more effective policy to reduce the burden on mentally ill patients.

## Background

Globally, major depressive disorder is an increasingly common disease with more than 300 million people suffering from it, according to the World Health Organization [1]. The one year incidence of major depression is approximately 5%, but can vary up to 10% in western European countries [2]. Moreover, the one year incidence of mood disorders in the United States is 9.5%, while in Asian countries it is much lower (Japan: 2.9%; South Korea: 3.6%) [3–5]. As a result, both the prevalence and social costs of depression are increasing, posing an important problem. In 2000, depression was determined to be one of the biggest causes of non-fatal burden [6]. In Germany, the direct cost of depression, per person, increased to 783 EUR (893.17 USD) in 2016 from 458.9 EUR (523.47 USD) in 2013 [7, 8]. Additionally, in Sweden, the total burden of depression almost doubled from 1997 to 2005 [9], which has also been reported in the United States (from 173.2 billion USD in 2005 to 210.5 billion USD in 2010) [10]. The economic burden of depression in Switzerland is approximately 8 billion EUR (9.13 billion USD) [11]; however, this figure is relatively underestimated in East Asia, as the direct costs of depression were cited as 8.09 billion RMB (0.99 billion USD) in 2007 in China [12] and 180 billion JPY (1.63 billion USD) in 2005 in Japan [13].

In some instances of depression, there are no symptomatic improvements with standard treatments. When the response to at least two different classes of antidepressants for an adequate period of time and dose is poor, these patients are defined as having treatment resistant depression (TRD), which has an incidence of approximately 15–20% within cases of depression [14, 15]. TRD patients are known to frequently use antipsychotics, non-pharmaceutical treatments, and inpatient treatment [16] and are often associated with multiple episodes of depression, decreased quality of life, and increased mortality [17]. Moreover, from a social standpoint, the economic burden of TRD is increasing worldwide, which is becoming problematic [18]. Compared to non-TRD patients, costs of TRD have increased by 29.3, 81, and 57% in the United States [19], Brazil [20], and Japan [21], respectively. Although there are large differences in costs per country, there is a paucity of studies determining the burden of depression in South Korea. To our knowledge, in 2012, the only study of its kind was conducted, which found that in South Korea, the direct health care costs of depression were 152.6 million USD, while the morbidity costs comprised the largest portion of the global burden of depression [22].

Through a national cohort, we have recently uncovered the epidemiology and characteristics of TRD in South Korea. We found that the incidence of TRD in Korea was 11.5 per 100 people in 2012. TRD accounted for roughly 0.08% of the total adult population and 4.17% of patients with depression. TRD patients required a longer duration of treatment and presented more comorbidities than non-TRD patients; however, the economic burden of depression in this region remains unclear [23]. Therefore, the aim of the present study was to investigate the economic burden of depression in South Korea. The first objective was to analyze the cost of depression using data from the National Health Insurance (NHI) in South Korea. The second objective was to compare the direct costs of TRD and non-TRD through several aspects such as risk of suicide, age, and the specialty of the doctor. We hypothesized that there would be higher costs associated with TRD than non-TRD, and that within the TRD group, there would be higher expenses due to the increased rates of suicide, age, and non-psychiatric care. Therefore, in the present study, by analyzing the differences in costs between TRD and non-TRD, we aimed to better understand the socioeconomic burden of TRD that would aid in future policy creation.

## Methods

### Data sources

In South Korea, there is a unique NHI that covers 98% of the population [24]. They developed a claiming system called the Health Insurance Review and Assessment Service (HIRA), which contains data from 90% of Koreans [25]. Moreover, researchers can request statistical data from HIRA to conduct studies [24]. In the present study, medical records and expenses via receipts were collected from HIRA from between January 1, 2011, to December 31, 2015. As no personal information was collected, no consent was required. The protocol of the present study was exempt from review by the Institutional Review Board (IRB) of Seoul National University Hospital and the Seoul National University College of Medicine (IRB number: 1611–056-807).

### Definitions of pharmaceutically treated depression (PTD) and TRD

PTD is characterized by successful treatment of depression with prescribed antidepressants. Therefore, the patients were placed in the PTD group when the diagnosis of depression and prescription of antidepressants were made within 30 days of each other, similar to the definition in studies using national registry data [21, 26]. In the PTD patients, the first day of antidepressant prescription was considered the index date and the PTD episode ended when the patient was no longer prescribed antidepressants for 120 days or more and no longer met diagnostic criteria. In cases of multiple PTD episodes in a single patient, only the first episode was assessed.

In this study, patients were considered to have TRD when two or more antidepressants were ineffective in treating their depression, same as the definition in previous studies. For the definition of TRD, most studies focused on the lack of response to drugs, and clinical definitions were virtually impossible in studies using national registry data [27]. Therefore, as in other studies in Japan, Taiwan, and the United States, TRD was defined as the subsequent administration of a third antidepressant prescription, and the end date of the second prescription was the index date of TRD [21, 26, 28]. Detailed criteria for the definitions of PTD and TRD have been described in a previous study publishied in 2019 [23].

### Medication regimen

The duration of medication was set to be 15 days in studies from Japan and 30 days in studies from the United States [21, 28]. One regimen was defined as the continuous use of one antidepressant or antipsychotic agent for more than 28 days during the PTD period in this study, based on a Taiwanese study [26]. This is because a 15-day period is too short to confirm the response to antidepressants [29]. The first regimen had to be started with antidepressants, while the second one could be started with another type of drug. Moreover, when the drug was discontinued for at least 30 days during the PTD period, it was considered to be the end of the regimen. Patients were considered to be in remission if at the end of the regimen, there had been no antidepressant or antipsychotic prescription for 120 days. A minimum dose criterion was set for antipsychotic regimens, but not for antidepressant regimens, because antipsychotics may be used for other purposes, such as sleep control or antidepressant effects [30]. The failure of one regimen was defined as a change to another regimen and when the same drug was used for more than 90 days, it was considered to be effective. Specific medication regimens and the minimal dose for drugs have been previously described [23].

### Study populations

Of the available medical records since 2011, data were collected from those who had been prescribed antidepressants more than once since 2012 and were over the age of 18 at the time of prescription. The present study excluded patients who had antidepressant prescriptions for at least 4 months after the start of the study, as we aimed to investigate new depressive episodes.

Subjects were excluded if they received an exclusionary diagnosis during the study period or in the case of death. Exclusionary diagnoses included psychotic (schizophrenia [F20], schizotypal disorder [F21], persistent delusional disorders [F22], acute and transient psychotic disorders [F23], schizoaffective disorder [F25], or other nonorganic psychotic disorders [F28]) and neurocognitive disorders (Alzheimer’s disease [F00], vascular dementia [F01], dementia in other diseases classified elsewhere [F02], or unspecific dementia [F03]). Additionally, manic episodes (F30) and bipolar affective disorder (F31) were excluded if they were the primary diagnosis; in South Korea, there are often cases where the diagnosis is made without a clear manic episode so mood stabilizers can be prescribed.

A portion of the PTD patients were prescribed antidepressants at the end of the observation period of the present study. As their episode was still ongoing, it was not possible to distinguish between non-TRD and TRD diagnoses; however, we recognized that TRD accounts for approximately 4% of cases, so we included them in the non-TRD group.

### Medical costs

In the present study, the direct medical costs were assessed based on costs of prescriptions and those reflected by receipts registered with HIRA; however, if patients visited several departments or hospitals on the same day, it was registered as one receipt in HIRA. Receipts generated at the hospital included the costs of the prescribed medication, counseling, and other psychological and blood tests that may have been prescribed by the doctor. As a result, if the medical expenses were calculated as a sum of the costs reflected by the receipts, it was more likely to be overestimated. Therefore, through the details of each receipt, the circumstances in which the receipt was issued could be inferred. For example, when the receipts with codes indicating depression were selected separately and the costs were calculated, the medical expenses related to depression could be estimated.

For each receipt, the amounts paid by the health insurance corporation and the individual were charged separately. Therefore, in the present study, the total amount was summed to measure the overall direct medical expenses.

Medical expenses were calculated with the receipts from January 1 to December 31, 2012. The insurance system in Korea had refund system for expenses that are considered overcharged. Refunds are usually made within 2 to 3 years of payment. Therefore, the cost in 2012, which is estimated to have ended the refund, could be the most accurate. The exchange rate to USD presented in this study was the rate in 2019.

### Variables

#### Depression and suicide codes

The diagnosis of major depressive disorder was defined as PTD when an antidepressant was prescribed, while the diagnostic codes were limited to F32, F33, and F34.1. Cases of suicide were defined when the patient received a diagnostic code containing “self-harm” and related to the trauma associated with the suicide attempt. The trauma associated with suicide attempts included all the codes related to laceration to the wrist (S60, S61, S69), falling off a structure (i.e., building, cliff; W13, W15, W19), hanging (W75, W76), drowning (W65–74), and drug intoxication (F10-F19, except for nicotine intoxication [F17]).

#### Variables for hospital visits

There are several types of hospitals and clinics in Korea and they are divided into primary, secondary, and tertiary hospitals. Primary medical clinics (i.e., public health centers and health clinics) provide comprehensive health care that integrates prevention and treatment and are limited to less than 30 beds. Secondary medical institutions are hospitals with more than four medical professionals and are defined by their size (30 to 100 beds). Finally, tertiary medical institutions are advanced general hospitals with more than 100 medical beds or more than nine medical subjects and residents in each department.

#### Definitions of hospitalization and emergency room visits

If a receipt was classified as a hospitalization, costs were associated with hospitalizations were assumed. Moreover, if a patient visited an emergency room in Korea, the “emergency medical care cost” code may be necessary. Therefore, it was possible to verify that the claim with the associated code was, in fact, related to an emergency room visit. In addition, the cost of visiting the emergency room included all causes of visit, without distinguishing the mental cause.

#### Definition of clinician specialty

In South Korea, only psychiatrists can request a psychiatric interview fee. Therefore, if patients ever had been billed a psychiatric interview charge, that receipt was deemed to be from a psychiatrist. The code for psychiatric interview charges included all codes related to family therapy, entertainment therapy, psychotic social work, and personal psychotherapy expenses (NN011–3, NN021–3, NN031–2, NN040, NN071–2, NN090, NN100, NN100020, NN111–4). The interview charge is differrent depending on the time of the psychiatrist has interviewed. And in 2018, the insurance system in Korea has changed so that the difference in interview costs over time becomes even greater. Therefore, shortening the interview with the psychiatrist reduced the patient’s burden.

### Statistical analysis

All continuous variables were presented as mean, standard deviation, median, and interquartile range, and all categorical variables were presented as numbers or percentages. The characteristics and cost differences between non-TRD and TRD were compared by the Wilcoxon rank sum test. SAS Enterprise Guide (version 6.1; SAS Institute, Cary, NC, USA) was used for statistical analysis and *p*-values less than 0.05 were considered statistically significant.

## Results

**Fig. 1:**
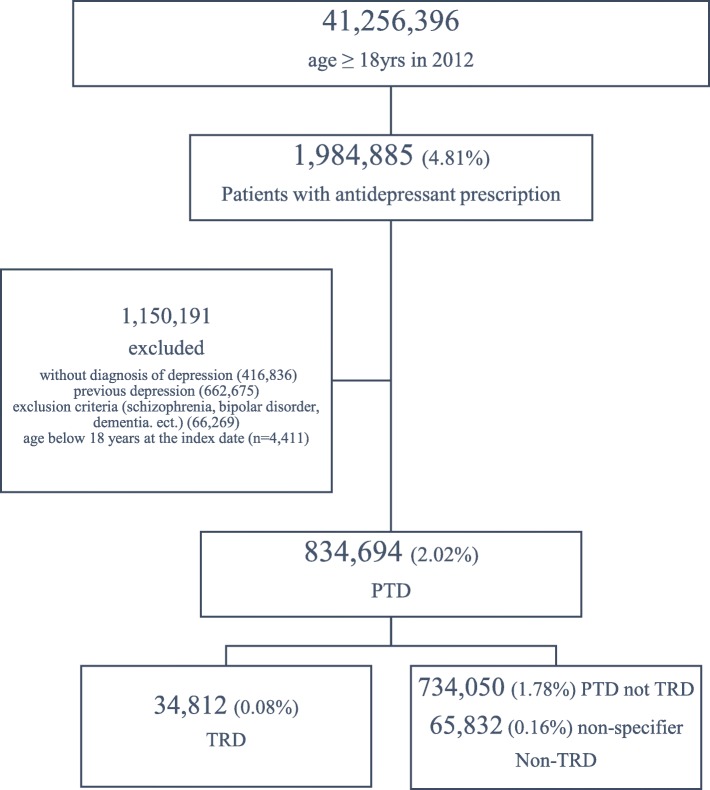
Patient Selection Flow

There were a total of 41,256,396 Koreans over 18 years of age in 2012, of which 4.81% were prescribed antidepressants and approximately half of those patients were diagnosed with depression. Moreover, 834,694 people (2.02% of the total population) were considered to have PTD, while 34,812 patients were diagnosed with TRD, accounting for 0.08% of the total population and 4.17% of PTD patients. There were subjects (65,832; 0.16% of the total population) who were unable to confirm the presence of TRD because the study was terminated during their follow-up; however, as the proportion of TRD to PTD is about 4%, they were included in the non-TRD group. Therefore, the total number of non-TRD patients was 799,882, accounting for approximately 1.94% of the total population (Fig. 1).

The average cost of medical expenses per visit for patients with PTD was 75,900 KRW (67.54 USD), and cost directly related to depression was 127,300 KRW (113.28 USD). Of the PTD patients, 21,613 (2.59%) had suicide codes, with associated costs estimated to be 62,800 KRW (55.87 USD), which is lower than the cost associated without suicide (77,800 KRW, 69.22 USD). The total medical costs associated with depression gradually increased as the type of hospital advanced (25,500 KRW, 22.58 USD in primary hospitals; 172,200 KRW, 152.46 USD in secondary hospitals; 178,700 KRW, 158.66 USD in tertiary hospitals). This pattern was also observed in the medical costs associated with depression codes. Psychiatrists found that the overall cost associated with PTD was consistent with the costs based on depression codes (96,100 KRW, 85.50 USD); however, in the group of patients treated by non-psychiatrists, the costs associated with depression were increased, compared to the total cost (71,500 KRW, 63.61 USD in total costs and 164,800 KRW, 146.62 USD with depression code) (Table 1).

**Table 1:** Cost per receipt of medical utilization in total PTD (1000 KRW, 0.89USD)

	Total cost	With depression code (*N* = 834,694)	With suicide code (*N* = 21,613)	Without suicide code (*N* = 813,081)
mean ± SD	75.9 ± 499.4	127.3 ± 748.2	62.8 ± 345.2	77.8 ± 518.0
median (Q1, Q3)	15.0 (11.5,34.3)	29.1 (13.7,46.3)	15.0 (11.8,32.7)	15.0 (14.0,34.6)
Type of hospital				
Primary	25.5 ± 58.5	34.2 ± 63.4	25.3 ± 57.2	25.5 ± 58.6
Secondary	172.2 ± 577.3	256.2 ± 706.3	159.1 ± 484.0	174.1 ± 589.7
tertiary	178.7 ± 944.5	259.9 ± 1284.6	140.0 ± 655.3	183.6 ± 975.5
Specialty of physician				
Psychiatry	96.1 ± 547.7	96.1 ± 547.7	108.3 ± 428.1	94.6 ± 560.8
Non-psychiatry	71.5 ± 488.3	164.8 ± 932.5	54.5 ± 327.1	74.1 ± 508.1
hospitalization	1761.1 ± 2614.9	2097.4 ± 2968.9	1358.1 ± 1759.2	1817.0 ± 2707.7
Outpatients visit	32.4 ± 67.9	38.4 ± 64.5	30.9 ± 58.2	32.7 ± 69.2

*PTD* pharmaceutically-treated depression, *SD* standard deviation, *Q1* first quartile, *Q3* third quartile

When analyzing the utilization of medical services, in TRD patients compared to non-TRD patients, more outpatient visits occurred in depressive episodes (95.2 versus 15.9), emergency room visits (4.7% versus 0.6%), hospitalizations (3.0 times per patient and 1.7 times per patient), and longer hospitalizations (63.4 days versus 28.9 days). The total medical costs per person for TRD and non-TRD patients were 6,610,500 KRW (5859.33 USD) and 1,273,000 KRW (1128.35 USD), respectively. Moreover, the ratio of TRD to non-TRD costs was 5.19. In the analysis of medical costs associated with the depression diagnosis, the TRD group incurred costs that were approximately 5 times higher than those in the non-TRD group. When analyzed in conjunction with suicide, it was confirmed that the suicide-related rate was higher in TRD patients than in non-TRD patients (12.95% compared to 2.14%). The total cost associated with TRD patients was 10,539,200 KRW (9343.26 USD), while non-TRD patients averaged 4,902,300 KRW (4346.01 USD), demonstrating that costs associated with TRD were roughly 2.15 times higher than non-TRD. Finally, costs unrelated to suicide were 5.05 times higher in TRD patients than non-TRD patients (6,026,000 KRW [5341.73 USD] versus 1,193,700 KRW [1058.15 USD]) (Table 2).

**Table 2:** Medical utilization and total cost between TRD and non-TRD (1000 KRW, 0.89USD)

		TRD (*N* = 34,812)	Non-TRD (*N* = 799,812)	*P*-value	TRD /non-TRD ratio
Clinical medical utilization					
Outpatients visits	mean ± SD	95.2 ± 102.9	15.9 ± 41.3		
	median (Q1, Q3)	64 (28,128)	3(1,11)		
ER visits number		0.7(4.7)	0.1(0.6)		
Hospitalization (N, %)		17,246(49.5)	149,901(18.7)		
Number of hospitalization (mean, SD)		3.0 ± 3.6	1.7 ± 1.8		
Days of hospitalization (mean, SD)		63.4 ± 145.0	28.9 ± 71.4		
Medical cost					
Total cost	mean, SD	6610.5 ± 11,126.7	1273.0 ± 4604.7	< 0.0001	5.19
	median (Q1,Q3)	3326.2(1288.67157.0)	142.0(42.9685.9)		
Related depression code	mean, SD	3461.6 ± 7670.8	697.7 ± 3059.4	< 0.0001	4.96
	median (Q1,Q3)	1384.3(543.93182.3)	70.4(26.2258.3)		
	**Patients with suicide code (N, %)**	4508 (12.95%)	17,105 (2.14%)		
Total cost	mean, SD	10,539.2 ± 14,061.1	4902.3 ± 9358.5	< 0.0001	2.15
	median (Q1,Q3)	6178.4(3187.711790.9)	2113.6(605.35414.7)		
Related depression code	mean, SD	5375.7 ± 9661.1	2138.5 ± 5714.6	< 0.0001	2.51
	median (Q1,Q3)	2464.5(1115.44927.5)	559.2(152.0,1923.7)		
	**Patients without suicide code (N)**	30,304 (87.05%)	782,777 (97.86%)		
Total cost	mean, SD	6026.0 ± 10,496.5	1193.7 ± 4411.2	< 0.0001	5.05
	median (Q1,Q3)	2955.8(1152.26458.3)	135.7(41.9628.3)		
Related depression code	mean, SD	3176.9 ± 7286.0	666.2 ± 2967.3	< 0.0001	4.77
	median (Q1,Q3)	1255.0(501.42947.4)	68.2(25.7242.2)		

*TRD* treatment-resistant depression, *SD* standard deviation, *Q1* first quartile, *Q3* third quartile, *ER* emergency room, *ECT* Electroconvulsive therapy

Upon further analysis of the costs of TRD, costs associated with TRD were highest in primary health care centers (5.15 times higher) than secondary and tertiary hospitals (3.45 times higher). The difference between prescriptions by psychiatrists and non-psychiatrists was similar (3.74–3.88 times), and the cost of prescriptions by psychiatrists were 0.57 and 0.59 times smaller in the TRD and non-TRD groups, respectively. The economic burden gap between TRD and non-TRD patients due to hospital admissions was minimal (1.72 times); however, the cost of outpatient visits was 5.93 times higher in the TRD group. Finally, in the TRD group, the total costs incurred by psychiatrists was lower than that of non-psychiatrists, whereas in the non-TRD group, the opposite result was obtained (Table 3).

**Table 3:** Total cost-subtype between TRD and non-TRD (1000 KRW, 0.89 USD)

	TRD	Non- TRD	P-value	TRD/non-TRD ratio
By Type of hospital				
1) Primary (N, %)	33,245 (95.50%)	598,848 (74.87%)		
1) Primary (mean, SD)	1872.1 ± 2951.2	363.6 ± 1384.6	< 0.0001	5.15
2) Secondary + tertiary (N, %)	31,610 (90.80%)	519,996 (65.01%)		
2) Secondary + tertiary (mean, SD)	5311.2 ± 11,023.8	1539.5 ± 5263.9	< 0.0001	3.45
By specialty of Physician (psychiatrists vs. non-psychiatrists)				
1) psychiatrists (N, %)	30,484 (87.57%)	295,638 (35.71%)		
1) psychiatrists (mean, SD)	2551.6 ± 5289.3	683.0 ± 2606.3	< 0.0001	3.74
2) non-psychiatrists (N, %)	34,085 (97.91%)	709,324 (88.68%)		
2) non-psychiatrists (mean, SD)	4469.5 ± 9520.2	1150.9 ± 4371.3	< 0.0001	3.88
Hospitalization, outpatients visit				
1) Hospitalization (N, %)	17,246 (49.54%)	149,901 (18.74%)		
1) Hospitalization (mean, SD)	6978.1 ± 12,539.9	4053.3 ± 7657.0	< 0.0001	1.72
2) Outpatients visit (N, %)	34,704 (99.69%)	770,279 (96.30%)		
2) Outpatients visit (mean, SD)	3163.3 ± 4379.6	533.2 ± 219.0	< 0.0001	5.93

*TRD* treatment-resistant depression, *SD* standard deviation

**Fig. 2:**
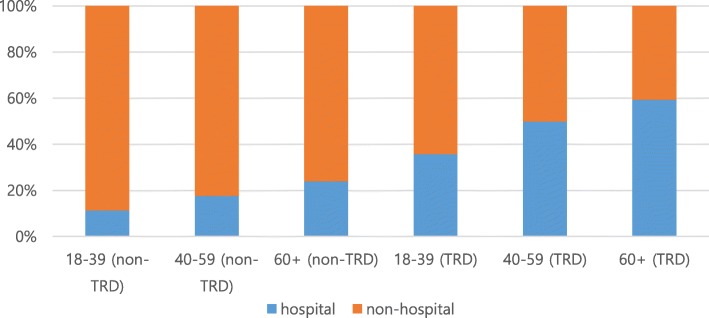
Age related hospitalization ratio between TRD and non-TRD

When costs were analyzed according to age, it was confirmed that the total cost increased in both TRD and non-TRD patients as age increased. The costs associated with depression were similar, except that middle-aged patients had the highest costs in the TRD group and for costs related to hospitalization (Table 4); however, when the ratios within each group were examined, the proportion of costs for hospitalization gradually increased as age increased, particularly within the TRD group (Fig. 2).

**Table 4:** Total cost analysis by age (1000 KRW, 0.89 USD)

	TRD	Non-TRD	P-value	TRD/non-TRD ratio
PTD patients aged 18–39 (N)	8294	164,113		
Total cost, won (mean, SD)	4186.4 ± 8013.9	621.9 ± 2707.2	< 0.0001	6.71
With depression code	2789.7 ± 5915.4	436.2 ± 2095.6	< 0.0001	6.40
With hospitalization (N, %)	2961 (35.70%)	18,530 (11.29%)		
With hospitalization (mean, SD)	5233.5 ± 10,970.8	2750.6 ± 6448.0	< 0.0001	1.90
PTD patients aged 40–59 (N)	15,157	330,818		
Total cost, won (mean, SD)	7010.9 ± 12,061.7	1151.0 ± 4621.8	< 0.0001	6.09
With depression code	3895.1 ± 838.9	677.4 ± 3157.4	< 0.0001	5.75
With hospitalization (N, %)	7545 (49.78%)	58,474 (17.68%)		
With hospitalization (mean, SD)	7690.2 ± 13,547.7	3895.6 ± 8070.3	< 0.0001	1.97
PTD patients aged 60+ (N)	11,361	304,951		
Total cost, won (mean, SD)	7846.0 ± 11,505.0	1755.9 ± 5293.5	< 0.0001	4.47
With depression code	3373.8 ± 776.5	860.4 ± 3363.1	< 0.0001	3.92
With hospitalization (N, %)	6740 (59.33%)	72,897 (23.90%)		
With hospitalization (mean, SD)	6947.3 ± 11,923.1	4511.0 ± 7556.0	< 0.0001	1.54

*TRD* treatment-resistant depression, *SD* standard deviation

## Discussion

The present study analyzed the medical costs associated with depression using the NHI, where the majority of the Korean population is enrolled. According to the HIRA survey, in 2012, PTD patients accounted for approximately 2% of the total population, and 4% of which progressed to TRD. Cost associated with depression for one hospital visit was 127,300 KRW (113.28 USD) in South Korea. A total of 834,694 PTD patients made an average of 19.2 outpatient visits. Based on that, the annual bill was calculated to be approximately 1.2 trillion KRW (1.07 billion USD) for outpatient visits and 294 billion KRW (261 million USD) for hospitalizations. The sum of outpatient and hospitalization costs was calculated, and total amount paid by PTD patients for 1 year was 1.494 trillion KRW (1.331 billion USD), which was substantially higher compared to other countries [7, 12] (supplementary Table 1). In the present study, it has been demonstrated that TRD costs more than non-TRD, which is consistent with previous results [18–21]. The cost burden of TRD was 1.72 to 5.93 times more than non-TRD, specifically in total medical costs, patients without a suicide code, primary hospitals, and outpatient visits. Results of this study are consistent with the TRD-to-non-TRD ratio obtained in studies from other countries. In a study conducted in Japan in 2017, the TRD-to-non-TRD ratio was approximately 3 [21]. In addition, results of the US study showed that the TRD-to-non-TRD ratio ranged from 2.7 to 5.0, although there were some differences depending on the type of medical use [28]. However, in a 2002 US study, the TRD group had six-fold higher admission costs and 19-fold higher depression-related costs than the non-TRD group [31]; however, previous studies have analyzed papers from 1995 to 2000, and as the prevalence of depression has increased in the last 15 years, the economic costs of depression have increased considerably. As the prevalence of depression rapidly increases, the cost of depression has a comparable increase, but it is possible that TRD has a smaller increase due to its low prevalence. Our previous study revealed that a longer duration of depressive episodes and presence of comorbidities were more frequent in the TRD group [23]. This might explain the higher cost in TRD.

Medical costs per receipt associated with suicide was lower than those without a suicide code; however, when considering groups treated by a psychiatrist, the costs associated with suicide code were higher. Additionally, the entire medical costs related to suicide were higher than the total medical costs in both the TRD and non-TRD groups. This could be explained by the differing group sizes and psychotherapy fees. Suicide codes were only observed in 2.59% of PTD patients (834,694 versus 21,613 with a suicide code). Patients with suicide codes who were treated by a non-psychiatrist may not be charged a psychotherapy fee, which could yield the above results.

In the present study, differences in costs according to the prescribing doctor’s specialty were found to be opposite in the TRD and non-TRD groups, which may be attributed to Korea’s claim system. In Korea, only psychiatrists can charge for psychotherapy; therefore, when a patient with depression visits a hospital, psychiatrists are allowed to request an interview fee along with the medical prescription, but non-psychiatrists can only prescribe medication for their patients. In cases of non-TRD, the difference in the number of interviews are reflected in our present results, as pharmacological treatments show better responses and a shorter duration of use than in TRD patients; however, because TRD can be difficult to treat with medication, it requires higher costs and interventions as these patients use more complex drugs for a longer period of time. Based on the results of the present study, it would be advantageous to refer to psychiatrists when patients do not respond to medications prescribed by non-psychiatrists.

There was an increase in the costs of depression by age; specifically, the cost of hospitalization gradually increased with age in the non-TRD group. However, in the TRD group, the cost associated with depression codes and hospitalizations was highest in middle-aged group. This result could be interpreted in a variety of ways. First, it is likely that the average PTD patient was of middle age. The proportion of hospitalization costs increased with age when the difference in the number of people was corrected for by assessing the percentage of the cost. Second, middle-aged patients are more likely to actively seek treatment, as this is a relatively stable occupational and economic age group, compared to other age groups. Third, depression in old age is often not diagnosed as depression because it often appears as other symptoms, such as lethargy, instead of exclusively depression [32]. This leads to higher medical expenses focused on the somatic symptoms not typically associated with depression, such as chest pain and abdominal discomfort. Fourth, psychiatric medication in elderly patients should be proceeded and monitored with caution, as the period for medication adjustments is longer and the adverse effects due to the medication are more frequent [33–35]. Fifth, depression in adolescents is often difficult to diagnose due to hostile behaviors, such as impulsivity, rather than depression [36].

The strength of this study is that it was a large, nationwide, cohort study of the majority of the Korean population. As a national registry was used, it was possible to exclude reduced reports of pharmacological prescriptions. Furthermore, the accuracy of the diagnosis was increased due to the use of the national registry. To the best of our knowledge, this is the first study to analyze the economic burden of depression in Korea from various perspectives. Analyses of the direct medical costs due to depression have occurred that have assessed various aspects, and the redistributive aspects of social property should be considered. Therefore, to reduce the economic burden of depression in Korea, it can be concluded that psychiatrists should be more involved in the treatment at primary care and outpatient centers, which provide the majority of the expenses.

However, there are several limitations to the present study. First, in this paper, the cost was calculated based on the 2012 data. The current economic trends may differ from those 8 years before. As described in the Methods section, several years later, excessive medical expenses in Korea could be reimbursed with insurance. In this study, we analyzed data from 2012, when the refund process was completed, and cost was fixed. However, in 2018, there was a big change in the system of psychiatric interview charging. This has had a large impact on the cost burden of psychiatric patients, but the cost study has practical limitations because of the refund system. Compared to the economic burden in this study, if the economic burden due to changes in 2018 is analyzed in the future, the impact of those changes will be clearer. Second, although the definitions of PTD and TRD are limited, it is difficult to apply them in a retrospective evaluation of national registry data. In addition, it is difficult to determine if the drugs prescribed based on the definitions of PTD and TRD were actually administered, possibly leading to an overestimated cost. However, it is possible to overcome this limitation because these drugs are rarely prescribed once but often repeatedly for a certain period of time, and repeated doses of unused medicines are rare. Third, the medication regimen used was in 4-week increments, but this may not reflect a realistic prescription. There is a tendency to add antipsychotics to a mood stabilizer regimen in Korea [37]; however, our results were confirmed with a subsequent analysis comparing 2 week and 6 week regimens. In this analysis, there was a slight difference, but it did not affect the interpretation (Supplementary Table 2). Fourth, Korea’s billing system is different from other countries, and depending on the diagnosis, the amount that the medical staff can claim varies. Therefore, it is difficult to judge whether the diagnostic code registered in HIRA is a diagnosis that accurately reflects the condition of a patient or if a diagnosis is necessary for a charge request. For example, there is a limited number of patients for whom a psychiatrist can charge a fee; therefore, receipts that do not indicate costs for psychiatric interviews may still have been issued by a psychiatrist. In this case, the cost of medical care from a psychiatrist may be underestimated. However, in many cases this is untrue; the present study included 98% of the Korean population and could be protected from this error because they were followed for roughly one year. Fifth, for patients who were considered to have PTD until the end of the study period, it was unclear whether they were non-TRD or TRD; therefore, we included these patients in the non-TRD group. We classified 7.9% of patients who were prescribed antidepressants at the end of the observation period as non-TRD, which could lead to statistical error. In particular, it is possible that some of the TRD patients should have been included as non-TRD patients, which may result in an underestimation in the difference between these groups. Patients who were unable to clarify whether they were TRD or non-TRD were differentiated and their PTD characteristics were compared with non-TRD and TRD groups, confirming they were generally similar to the non-TRD group (Supplementary Table 3). Finally, in the present study, we compared the direct medical costs through the analysis of the medical receipts registered in the NHI; however, the economic burden of depression is not exclusively the direct medical costs. Indirect medical expenses, such as leave and unemployment due to depression, can also be an economic burden. Moreover, according to a recent systemic review of 24 studies, indirect medical costs are twice as much as direct medical costs [38]. It is a methodological limit of the present study that only the direct costs were assessed; therefore, future studies should attempt to elucidate some of the indirect medical costs associated with depression. Alternatively, using data registered in the country prevented recall bias and allowed for the confirmation of the correct amount without error.

In the present study, we analyzed the cost differences between TRD and non-TRD and confirmed that the primary difference was whether they received medical treatment from a psychiatrist. To better understand this difference, it is crucial to analyze data about the characteristics of depression, sex difference, and whether the first intervention was by a psychiatrist. It would be valuable if the present study was repeated annually and compared with other countries with a different insurance system. Additionally, further investigation is needed to better understand the increasing hospitalizations with age to reduce hospitalization costs in the elderly.

## Conclusions

Through the present study, it was determined that the average cost for one visit for a patient with depression in Korea was 127,300 KRW (113.28 USD), which is quite socially significant. Patients with TRD are burdened with higher medical costs than are non-TRD patients, especially when including increasing age, visiting primary care clinics, and outpatient care as variables. The costs associated with depression were also lower when visiting a psychiatrist, which suggests that active treatment interventions by psychiatrists in the early stages of depression and establishing more effective policies, such as an early-refer program to psychiatrists, may reduce the direct medical care costs for depression. More in-depth analyses of the costs of depression should be performed to help establish a better political health plan.

## Supplementary information

**Additional file 1 Supplementary Table 1.** Medical utilization of total PTD patients. **Supplementary Table 2.** Medical utilization and cost comparing by regimen. **Supplementary Table 3.** Clinical characteristics and cost analysis by TRD, PTD was not TRD, TRD unknown group

## Data Availability

Requests for more detailed information regarding the study can be addressed to the corresponding author. All data used in this study were obtained from http://opendata.hira.or.kr. Access to this database is open to anyone.

## Abbreviations

PTD: Pharmaceutically treated depression
TRD: Treatment-resistant depression
HIRA: The Health Insurance Review and Assessment Service
KRW: Korean won
USD: US dollar
NHI: The National Health Insurance
IRB: The Institutional Review Board
SD: Standard deviation
Q1: First quartile
Q3: Third quartile
ER: Emergency room
ECT: Electroconvulsive therapy
