# Supplementary material for: Cost analysis of depression using the national insurance system in South Korea: a comparison of depression and treatment-resistant depression
Source: BMC Health Serv Res. 2020 Apr 6;20:286. doi: 10.1186/s12913-020-05153-1 (PMC7137426; doi:10.1186/s12913-020-05153-1)
Supplement: Supplementary file 1 — Additional file 1 Supplementary Table 1. Medical utilization of total PTD patients. Supplementary Table 2. Medical utilization and cost comparing by regimen. Supplementary Table 3. Clinical characteristics and cost analysis by TRD, PTD was not TRD, TRD unknown group [file 12913_2020_5153_MOESM1_ESM.docx]

Supplementary table 1. Medical utilization of total PTD patients

| PTD (N =834,694) | |
| --- | --- |
| PTD duration, days (mean, SD) | 152.3(303.6) |
| PTD duration, days (median, (Q1,Q3)) | 28(7,123) |
| Outpatients visits (mean, SD) | 19.2(48.3) |
| Outpatients visits (median, (Q1,Q3)) | 4(1,14) |
| ED visits (mean, SD) | 0.1(1.1) |
| ED visits (median, (Q1,Q3)) | 0(0,0) |
| Use of ECT |  |
| -Yes | 17(0.0) |
| Hospitalization |  |
| -Yes | 167147(20.0) |
| No. of hospitalization (mean, SD) | 1.8(2.1) |
| No. of hospitalization (median, (Q1,Q3)) | 1(1,2) |
| Days of hospitalization (mean, SD) | 32.4(82.8) |
| Days of hospitalization (median, (Q1,Q3)) | 12(5,29) |

*PTD* pharmaceutically-treated depression, *SD* standard deviation, *Q1* first quartile, *Q3* third quartile, *ER* emergency room, *ECT* Electroconvulsive therapy

Supplementary table 2. Medical utilization and cost comparing by regimen

|  | 14 day regimen | | 28 day regimen | | 42 day regimen | |
| --- | --- | --- | --- | --- | --- | --- |
|  | TRD | PTD that was not TRD | TRD | PTD that was not TRD | TRD | PTD that was not TRD |
|  | (N =55,151) | (N =718,598) | (N =34,812) | (N =734,050) | (N =22,400) | (N =742,589) |
| PTD duration, days (mean, SD) | 603.0(463.8) | 81.4(155.9) | 713.3(458.0) | 85.7(161.3) | 799.6(444.9) | 89.2(166.2) |
| PTD duration, days (median, (Q1,Q3)) | 447(193,1098) | 21(7,80) | 623(288,1180) | 21(7,86) | 789(383,1232) | 22(7,90) |
| Outpatients visits (mean, SD) | 81.9(96.8) | 10.3(23.8) | 95.2(102.9) | 11.0(25.1) | 105.4(105.4) | 11.4(26.2) |
| ER visits (mean, SD) | 0.6(3.8) | 0.1(0.4) | 0.7(4.7) | 0.1(0.4) | 0.8(2.5) | 0.1(0.5) |
| Use of ECT |  |  |  |  |  |  |
| -Yes | 10(0.0) | 2(0.0) | 9(0.0) | 2(0.0) | 7(0.0) | 2(0.0) |
| Hospitalization |  |  |  |  |  |  |
| -Yes | 25409(46.1) | 114094(15.9) | 17246(49.5) | 119370(16.3) | 11679(52.1) | 122633(16.5) |
| No. of hospitalization (mean, SD) | 2.8(3.4) | 1.5(1.5) | 3.0(3.6) | 1.5(1.5) | 3.1(3.7) | 1.6(1.6) |
| Days of hospitalization (mean, SD) | 56.6(131.1) | 24.8(54.3) | 63.4(145.0) | 25.4(55.7) | 69.3(156.8) | 25.9(56.9) |
| **Total cost, won (mean, SD)** | 66814.8(387300) | 85244.2(588239.1) | 67770.8(385317.8) | 83998.1(578246.1) | 68375.7(375076.6) | 83421.0(573077.4) |
| **Total cost, won (median, (Q1,Q3))** | 16410(11810,37240) | 14800(11540,32600) | 16720(11830,38310) | 14830(11540,32780) | 17350(11980,39570) | 14850(11540,32960) |
| - By Type of hospital |  |  |  |  |  |  |
| 1) Primary hospital (mean, SD) | 26677.3(59683.3) | 24845.2(59097.4) | 27114.7(62341.4) | 24899.1(58639.5) | 27550.8(58806.1) | 24962.0(60394.5) |
| 2) Secondary hospital (mean, SD) | 169647.9(529760.7) | 176837.1(605975.8) | 174321.0(537550.9) | 175830.2(600791.8) | 178832.3(539783.6) | 175823.9(599229.8) |
| 3) Tertiary hospital (mean, SD) | 147020.2(728081.9) | 209730.6(1114983.9) | 143569.5(708187.0) | 207254.6(1101094.0) | 141007.4(679332.9) | 205122.5(1090743.0) |
| - By specialty of Physician (psychiatrists vs. non-psychiatrists) |  |  |  |  |  |  |
| 1) psychiatrists (mean, SD) | 91321.6(417304.4) | 101745.3(668695.7) | 93365.9(406038.7) | 100181.8(652134.8) | 94181.0(395518.2) | 99610.2(640871.1) |
| 2) non-psychiatrists (mean, SD) | 59108.6(377043.4) | 82300.8(572650.6) | 59449.7(377963.9) | 80984.3(563365.6) | 59292.8(367180.1) | 80340.9(559196.5) |
| -Hospitalization, outpatients visit |  |  |  |  |  |  |
| 1) Hospitalization (mean, SD) | 1511683.6(2037036.5) | 1861730(2908446.4) | 1503152.2(2002243.2) | 1843138.2(2874384.1) | 1474860.3(1913366.8) | 1835093.9(2856454.2) |
| 2) Outpatients visit (mean, SD) | 32454.4(63740) | 31873.3(68008.7) | 33110.6(65569.3) | 31794.0(67436.4) | 33698.9(62583.8) | 31820.5(68460.8) |
| **Cost related with depression, won (mean, SD)** | 109264.9(565140.9) | 141423.6(859268.2) | 110932.1(555714.6) | 139394.3(846682.6) | 110291.0(530547.1) | 138648.2(840608.0) |
| **Cost related with depression, won (median, (Q1,Q3))** | 35120(24430,51590) | 26120(12890,42590) | 36270(24870,54250) | 26370(12900,42730) | 37230(25410,56130) | 26640(13050,43000) |
| - By Type of hospital |  |  |  |  |  |  |
| 1) Primary hospital (mean, SD) | 38582.9(61838.5) | 31502.7(66401.9) | 39921.8(65616.8) | 31743.6(65485.7) | 40743.5(66058.5) | 31982.8(65591.5) |
| 2) Secondary hospital (mean, SD) | 274389.2(652599.9) | 251080.7(735353.5) | 279403.9(653592.9) | 251968.8(732097.2) | 284843.5(647317.2) | 253456.5(732302.3) |
| 3) Tertiary hospital (mean, SD) | 205733.1(983055.9) | 307102.5(1487500.1) | 196395.0(937326.4) | 304708.7(1475719.2) | 189270.3(883582.0) | 301659.7(1464386.9) |
| - By specialty of Physician (psychiatrists vs. non-psychiatrists) |  |  |  |  |  |  |
| 1) psychiatrists (mean, SD) | 91321.6(417304.4) | 101745.3(668695.7) | 93365.9(406038.7) | 100181.8(652134.8) | 94181.0(395518.2) | 99610.2(640871.1) |
| 2) non-psychiatrists (mean, SD) | 164081.4(871268.6) | 171724.3(979076.1) | 168727.8(882093.6) | 171745.8(977306.7) | 170098.4(860911.3) | 172356.5(979631.1) |
| -Hospitalization, outpatients visit |  |  |  |  |  |  |
| 1) Hospitalization (mean, SD) | 1813636.6(2304769.5) | 2150959.7(3226606.7) | 1793066.5(2234757.9) | 2136900.6(3198149.6) | 1741418.7(2108395.2) | 2132453.1(3182783.3) |
| 2) Outpatients visit (mean, SD) | 41844.8(49407.7) | 35449.6(63746.7) | 43358.2(48703.3) | 35507.8(62486.8) | 44602.6(48365.0) | 35647.6(61903.7) |

*TRD* treatment-resistant depression *, PTD* pharmaceutically-treated depression, *SD* standard deviation, *Q1* first quartile, *Q3* third quartile, *ER* emergency room, *ECT* Electroconvulsive therapy

Supplementary table 3. Clinical characteristics and cost analysis by TRD, PTD was not TRD, TRD unknown group

|  | **TRD**  **(N =34,812)** | **PTD that was not TRD**  **(N =734,050)** | **TRD status unknown**  **(N =65,832)** |
| --- | --- | --- | --- |
| PTD duration, days (mean, SD) | 713.3(458.0) | 85.7(161.3) | 598.5(593.7) |
| Outpatients visits (mean, SD) | 95.2(102.9) | 11.0(25.1) | 71.2(101.9) |
| ED visits (mean, SD) | 0.7(4.7) | 0.1(0.4) | 0.4(1.3) |
| Use of ECT (N, %) | 9(0.0) | 2(0.0) | 6(0.0) |
| Hospitalization (N, %) | 17246(49.5) | 119370(16.3) | 30531(46.4) |
| No. of hospitalization (mean, SD) | 3.0(3.6) | 1.5(1.5) | 2.2(2.6) |
| Days of hospitalization (mean, SD) | 63.4(145.0) | 25.4(55.7) | 42.5(112.6) |
| **Total cost, won (mean, SD)** | 67770.8(385317.8) | 83998.1(578246.1) | 67506.8(416821.5) |
| **Total cost, won (median, (Q1,Q3))** | 16720(11830,38310) | 14830(11540,32780) | 14880(11770,33680) |
| - By Type of hospital |  |  |  |
| 1) Primary hospital (mean, SD) | 27114.7(62341.4) | 24899.1(58639.5) | 25378.3(55181.7) |
| 2) Secondary hospital (mean, SD) | 174321.0(537550.9) | 175830.2(600791.8) | 163138.7(556637.4) |
| 3) Tertiary hospital (mean, SD) | 143569.5(708187.0) | 207254.6(1101094.0) | 152916.6(774164.4) |
| - By specialty of Physician |  |  |  |
| 1) psychiatrists (mean, SD) | 93365.9(406038.7) | 100181.8(652134.8) | 92141.3(485016.5) |
| 2) non-psychiatrists (mean, SD) | 59449.7(377963.9) | 80984.3(563365.6) | 62719.0(402052.7) |
| -Hospitalization, outpatients visit |  |  |  |
| 1) Hospitalization (mean, SD) | 1503152.2(2002243.2) | 1843138.2(2874384.1) | 1772501.1(2359385.8) |
| **Cost related with depression, won (mean, SD)** | 110932.1(555714.6) | 139394.3(846682.6) | 114751.5(654381.9) |
| **Cost related with depression, won (median, (Q1,Q3))** | 36270(24870,54250) | 26370(12900,42730) | 29410(13490,47060) |
| - By Type of hospital |  |  |  |
| 1) Primary hospital (mean, SD) | 39921.8(65616.8) | 31743.6(65485.7) | 34791.3(56217.5) |
| 2) Secondary hospital (mean, SD) | 279403.9(653592.9) | 251968.8(732097.2) | 248040.5(677986.6) |
| 3) Tertiary hospital (mean, SD) | 196395.0(937326.4) | 304708.7(1475719.2) | 219255.9(1086540.8) |
| - By specialty of Physician |  |  |  |
| 1) psychiatrists (mean, SD) | 93365.9(406038.7) | 100181.8(652134.8) | 92141.3(485016.5) |
| 2) non-psychiatrists (mean, SD) | 168727.8(882093.6) | 171745.8(977306.7) | 144466.3(824786.7) |
| -Hospitalization, outpatients visit |  |  |  |
| 1) Hospitalization (mean, SD) | 1793066.5(2234757.9) | 2136900.6(3198149.6) | 2252239.6(2796149.4) |
| 2) Outpatients visit (mean, SD) | 43358.2(48703.3) | 35507.8(62486.8) | 40288.4(77850.7) |

*TRD* treatment-resistant depression ***,*** *PTD* pharmaceutically-treated depression, *SD* standard deviation, *Q1* first quartile, *Q3* third quartile, *ER* emergency room, *ECT* Electroconvulsive therapy, *No* number
